# 超高效液相色谱-串联质谱法准确测定食品中非法添加的3种新型降压药

**DOI:** 10.3724/SP.J.1123.2024.02025

**Published:** 2024-10-08

**Authors:** Juzhou ZHANG, Jing LI, Pingping ZHANG, Mei YANG, Di ZHOU

**Affiliations:** 安徽省食品药品检验研究院, 国家农副加工食品质量检验检测中心, 安徽 合肥 230051; Anhui Provincial Institute for Food and Drug Control, China National Center for Quality Inspection and Test of Agricultural-Avocation Processed Food, Hefei 230051, China

**Keywords:** 超高效液相色谱-串联质谱法, 降压药, 阿齐沙坦, 坎地沙坦酯, 拉西地平, 食品, ultra-high performance liquid chromatography-tandem mass spectrometry (UHPLC-MS/MS), antihypertensive drugs, azilsartan, candesartan cilexetil, lacidipine, foods

## Abstract

为解决食品中非法添加化学降压药并以此宣称具有降压功能的食品安全问题,本研究基于超高效液相色谱-串联质谱(UHPLC-MS/MS)技术,建立了一种同时测定压片糖果、固体饮料、代用茶、茶饮料、饼干、果冻、配制酒和保健食品(口服液、茶剂、片剂、硬胶囊、软胶囊)等12种典型食品基质中阿齐沙坦、坎地沙坦酯和拉西地平等3种新型降压药的分析方法。样品以0.2%(v/v)甲酸乙腈提取,选择性采用QuEChERS净化,再用水稀释,经聚四氟乙烯膜过滤,以5 mmol/L甲酸铵水溶液-乙腈为流动相,通过Agilent Eclipse Plus RRHD C_18_色谱柱(50 mm×2.1 mm, 1.8 μm)进行分离,在电喷雾正离子扫描(ESI^+^)、多反应监测(MRM)模式下进行测定,基质匹配外标法定量。方法学研究表明,3种降压药在各自的线性范围内线性关系良好,相关系数(*r*^2^)均大于0.996;拉西地平的检出限(LOD)为0.02 mg/kg,定量限(LOQ)为0.04 mg/kg,阿齐沙坦和坎地沙坦酯的LOD均为0.01 mg/kg, LOQ均为0.02 mg/kg;对12种基质进行低、中、高3个水平的加标试验(*n*=6), 3种降压药的平均回收率为86.6%~107.5%,相对标准偏差(RSD)为1.1%~10.9%。该方法简单、快速、灵敏、准确,适用于食品中阿齐沙坦、坎地沙坦酯、拉西地平的同时测定,解决了这3种药物在食品基质中检测方法缺失的难题,可为监管部门提供技术支撑。

## Abstract (en)

Effective strategies are required to address food safety issues related to the illegal addition of antihypertensive drugs to food and claims of antihypertensive function. In this study, a novel ultra-high performance liquid chromatography-triple-quadrupole mass spectrometry (UHPLC-MS/MS) method was developed for the simultaneous determination of three antihypertensive drugs (azilsartan, candesartan cilexetil, and lacidipine) in 12 food matrices (pressed candies, solid beverages, alternative teas, tea drinks, biscuits, jellies, mixed liquors, oral liquids, medicinal teas, tablets, hard capsules, and soft capsules). Initially, mass spectrometry parameters, such as the collision energies of the three antihypertensive drugs, were optimized. Subsequently, the response intensities and chromatographic separation conditions of the three drugs in different mobile phases were compared. In addition, to enhance the recoveries, various extraction solvents and purification methods, including solid-phase extraction (SPE) columns and the QuEChERS technique, were investigated. In the developed method, sample determination involved three steps. First, the sample was extracted using 0.2% (v/v) formic acid in acetonitrile and then filtered using high-speed centrifugation, in addition, the extracted solution of alternative tea and medicinal tea was purified using the QuEChERS technique. Second, the supernatant was diluted with water, and filtered through a 0.22 μm polytetrafluoroethylene (PTFE) membrane. Finally, the analytes were separated on an Agilent Eclipse Plus RRHD C_18_ column (50 mm×2.1 mm, 1.8 μm) using a 5 mmol/L ammonium formate aqueous solution and acetonitrile as the mobile phases under gradient elution conditions and then detected using UHPLC-MS/MS with positive electrospray ionization (ESI) in the multiple reaction monitoring (MRM) mode. Quantitative analysis was performed using a matrix-matched external standard method. Methodological validation showed good linear relationships for all three antihypertensive drugs in the investigated concentration ranges, with correlation coefficients (*r*^2^) greater than 0.996. The limit of detection (LOD) and limit of quantification (LOQ) of lacidipine were 0.02 mg/kg and 0.04 mg/kg, respectively, whereas those of the other two drugs were 0.01 mg/kg and 0.02 mg/kg, respectively. In the 12 food matrices, the average recoveries of the drugs ranged from 86.6% to 107.5% with relative standard deviations (RSDs) of 1.1%-10.9% (*n*=6) at low, medium, and high spiked levels. Furthermore, this method was successfully applied to the analysis of real food samples. Overall, the newly developed method is simple, rapid, sensitive, accurate, and suitable for the qualitative and quantitative determination of antihypertensive drugs in different food matrices. This work could provide technical support for food safety agencies in implementing measures against the illegal addition of antihypertensive drugs to food.

随着《“健康中国2030”规划纲要》等政策及健康知识的推广,人们对身体的亚健康状态逐渐具备科学的认知,养生保健理念日益增强,在不断升级的消费诉求下,市场上涌现了大量降血压、降血脂、提高免疫力等具有功能声称的食品^[1]^。为牟取暴利,一些商家通过非法添加降压类药物来实现产品宣称的“奇效”,添加的产品类型从保健食品日趋转向普通食品,添加的药物种类和剂量也存在随意性和不确定性^[2,3]^,严重危害食用者的生命健康,国家市场监管总局已将食品中非法添加降压、降糖、降脂等物质列为重点监管对象。

降压药的种类繁多,阿齐沙坦和坎地沙坦酯属于血管紧张素Ⅱ受体拮抗剂,拉西地平属于钙拮抗剂^[4⇓⇓⇓-8]^,我国已批准这3种药物作为药品管理,其中阿齐沙坦是2021年在我国批准上市的新药,未被《中国药典》收载^[8]^,临床上多用于治疗高血压症,但在食品基质中发现此类药物属于不法商家为躲避监管而非法添加的新药。当前对这3种药物的研究主要集中在动力学机理、药物代谢、含量检测、有关物质及非法添加等方面,基质主要为药片、药剂、中成药、血浆和指印等^[9⇓⇓⇓⇓⇓⇓-16]^,而对于普通食品和保健食品中的检测方法鲜有报道,已发布的补充检验方法标准^[17⇓⇓⇓-21]^也未涵盖这3种药物。因此,针对食品基质亟待开展检测方法研究。

目前,已报道的降压药检测方法主要有薄层色谱法^[20]^、高效液相色谱法(HPLC)^[20,21]^、微乳液相色谱法(MELC)^[22]^、HPLC-串联质谱法(MS/MS)^[17⇓⇓⇓-21,23,24]^、HPLC-四极杆-高分辨质谱法^[25,26]^等。其中液相色谱法多应用于有关物质、含量检测等方面,高分辨质谱侧重于药物代谢、代谢物鉴定及非靶向筛选,HPLC-MS/MS以其具有更高的灵敏度、更强的专属性及抗干扰能力而被非法添加研究广泛采用。相关研究的基质多为药品或保健食品,阳性样品的目标分析物含量往往相对较大,前处理主要采用有机溶剂直接萃取或稀释法;对于普通食品,样品基质繁杂多样,宁霄等^[3]^对乳粉基质中的非法添加药物采取QuEChERS方法有效去除了基质中的脂质。QuEChERS方法操作简单、便捷,能有效去除部分色素、多糖、脂肪酸及其他有机酸等^[27]^,适合多样化、大批量样品的检测。

本工作针对目前宣称具有降压功能的压片糖果、固体饮料以及可能添加此类降压药的12种食品基质,依据不同形态样品基质的复杂程度,选择性采用QuEChERS方法净化,建立了超高效液相色谱-串联质谱(UHPLC-MS/MS)测定阿齐沙坦、坎地沙坦酯和拉西地平这3种降压药的方法,该方法操作简便、快速、准确,并将食品基质从重点聚焦的保健食品延伸至逃避监管的普通食品,为这3种新型非法添加降压类药物在普通食品基质中的检测提供了新方法,以期为食品安全监管及稽查执法提供技术支撑。

## 1 实验部分

### 1.1 仪器、试剂和材料

Agilent 1290-6495超高效液相色谱-串联质谱仪(美国Agilent公司), XP205电子天平(瑞士Mettler Toledo公司), S300H超声波清洗器(德国Elma公司), 3K15离心机(德国Sigma公司), MS3 Basic涡旋仪(德国IKA公司), Milli-Q超纯水机(美国Millipore公司)。

阿齐沙坦、坎地沙坦酯和拉西地平的纯度均≥99%,购自天津阿尔塔公司。

乙腈(ACN)和甲醇(色谱纯,美国Thermo Fisher公司),甲酸(FA)和甲酸铵(色谱纯,美国ACS公司),无水硫酸镁(分析纯,国药集团), C_18_吸附剂(上海安谱实验科技股份有限公司)。

样品:压片糖果(水蜜桃味清口含片)、代用茶(桑葚枸杞菊花茶)、固体饮料(甜橙味果珍)、茶饮料(红茶饮料)、饼干(番茄味香酥薄饼)、果冻(酵素果冻)、配制酒(青梅原味酒)、茶剂(罗布麻茶)、口服液(核苷酸绞股蓝口服液)、片剂(多种维生素矿物质片)、硬胶囊(西洋参纳豆胶囊)、软胶囊(辅酶Q10维生素E软胶囊)及用于实际样品检测的208批样品大部分由监督抽检部门提供,少数从电商和实体店铺购买。

### 1.2 溶液配制

标准储备液:分别准确称取标准品各10 mg(精确至0.01 mg),用甲醇溶解并分别定容于10 mL容量瓶中,配制成质量浓度均为1 g/L的标准储备液。-20 ℃避光保存。

混合标准中间液:分别准确吸取适量标准储备液,用甲醇稀释,配制成阿齐沙坦和坎地沙坦酯质量浓度为10 mg/L、拉西地平质量浓度为20 mg/L的混合标准中间液。-20 ℃避光保存。

空白基质溶液:取不含目标分析物的空白样品,按照1.3节条件进行前处理,获取空白基质溶液。

基质匹配混合标准工作液:准确吸取适量混合标准中间液,用空白基质溶液逐级稀释配制成系列质量浓度的基质匹配混合标准工作液。

### 1.3 样品前处理

对于固体和半固体试样(压片糖果、代用茶、固体饮料、饼干、果冻、茶剂、片剂、硬胶囊、软胶囊),取适量充分粉碎均质后,称取1 g(精确至0.001 g)试样于50 mL具塞刻度离心管中,准确加入25 mL 0.2%(v/v)甲酸乙腈溶液,涡旋混合,超声提取10 min, 6000 r/min离心4 min,准确吸取1.0 mL上清液(若样品为代用茶、茶剂时,则取1.5 mL上清液,加入30 mg C_18_吸附剂和100 mg无水MgSO_4_,涡旋振荡30 s,以8000 r/min离心4 min,再吸取1.0 mL上清液),用水稀释定容至2 mL,涡旋混匀,过0.22 μm亲水性聚四氟乙烯微孔滤膜,供UHPLC-MS/MS分析。

对于液体试样(口服液、茶饮料、配制酒),准确量取1.0 mL试样于25 mL容量瓶中,用0.2%甲酸乙腈溶液稀释至刻度,涡旋混匀。准确吸取1.0 mL样品溶液,用水稀释定容至2 mL,涡旋混匀,过0.22 μm亲水性聚四氟乙烯微孔滤膜,供UHPLC-MS/MS分析。

### 1.4 仪器分析条件

#### 1.4.1 色谱条件

Agilent Eclipse Plus RRHD C_18_色谱柱(50 mm×2.1 mm, 1.8 μm);柱温:35 ℃;流速:0.3 mL/min;进样量:2 μL;流动相A为5 mmol/L甲酸铵溶液,流动相B为乙腈,梯度洗脱程序:0~1.0 min, 5%B; 1.0~3.0 min, 5%B~98%B; 3.0~4.5 min, 98%B; 4.5~4.6 min, 98%B~5%B; 4.6~6.0 min, 5%B。

#### 1.4.2 质谱条件

离子源:电喷雾离子源(ESI);扫描方式:正离子扫描;检测方式:多反应监测(MRM);毛细管电压:3000 V;干燥气温度:210 ℃;干燥气流速:16 L/min;雾化气压力:207 kPa(30 psi);鞘气温度:330 ℃;鞘气流速:11 L/min;碎裂电压:380 V。3种降压药的监测离子对及质谱参数见表1。

**表1:** 3种降压药的CAS号、分子式、保留时间及质谱参数

Compound	CAS No.	Molecular formula	Retention time/min	Adduct ion	Precursor ion (m/z)	Product ions (m/z)	CEs/eV
Azilsartan (阿齐沙坦)	147403-03-0	C_25_H_20_N_4_O_5_	2.48	[M+H]^+^	457.1	233.0^*^, 279.0	22, 12
Candesartan cilexetil (坎地沙坦酯)	145040-37-5	C_33_H_34_N_6_O_6_	3.28	[M+H]^+^	611.0	423.0^*^, 349.0	12, 38
Lacidipine (拉西地平)	103890-78-4	C_26_H_33_NO_6_	3.72	[M+H]^+^	456.1	400.0^*^, 354.0	16, 16

* Quantitative ion; CEs: collision energies.

## 2 结果与讨论

### 2.1 色谱条件的优化

#### 2.1.1 色谱柱的选择

从有机化合物的分配系数(即lg *P*)^[3]^来看,lg *P*值愈大,则疏水性愈强,即极性越小。阿齐沙坦、坎地沙坦酯和拉西地平的lg *P*值分别为4.4、7.0和4.5,极性相对较弱,但均不属于强疏水性化合物,可选择反相色谱柱进行分离。实验比较了Agilent Eclipse Plus RRHD C_18_(50 mm×2.1 mm, 1.8 μm)、Agilent Eclipse Plus RRHD C_18_(100 mm×2.1 mm, 1.8 μm)和Waters HSS T_3_(100 mm×2.1 mm, 1.8 μm)色谱柱的分离效果。在使用C_18_色谱柱时,目标分析物的响应强度和分离度均较好,但在Waters HSS T_3_色谱柱上坎地沙坦酯的响应强度明显下降,坎地沙坦酯与拉西地平的分离度也不如在其他两支色谱柱上好。可见,100 mm和50 mm的C_18_柱均满足实验要求,但由于长色谱柱的柱压更高,分析更耗时,故选择50 mm的C_18_色谱柱进行后续分析。

#### 2.1.2 流动相的选择

在ESI^+^下,甲酸水溶液可以为正离子扫描提供质子,提高母离子的离子化效率,甲酸铵等缓冲盐溶液虽然对离子化过程有抑制效应,但可以改变色谱保留行为,提高分离度。实验首先考察了不同体积分数(0.01%、0.02%、0.05%、0.1%、0.15%)的甲酸水溶液-乙腈体系对分离效果的影响。结果显示,3种目标分析物的响应强度均较高,使用0.01%甲酸水溶液-乙腈时,响应强度达到最高,但拉西地平的峰形对称性欠佳,且与坎地沙坦酯的峰重叠(图1a)。实验随即比较了10 mmol/L缓冲盐体系(甲酸铵溶液-乙腈、甲酸铵溶液-甲醇、乙酸铵溶液-乙腈、乙酸铵溶液-甲醇)的分离效果。结果发现,坎地沙坦酯和拉西地平被完全分离(图1b),但3种目标分析物的响应强度均有不同程度的降低,乙酸铵溶液使响应强度降低得更多,且甲醇为有机相时,拉西地平的峰前延严重。因此,实验选择甲酸铵溶液-乙腈体系并进一步对比了不同浓度(5、10、15、20 mmol/L)甲酸铵溶液的分离效果。结果表明,采用5 mmol/L甲酸铵溶液-乙腈时的峰形、响应强度和分离度均最优。为保持良好的分离度和提高响应强度,实验最后考察了含0.01%、0.02%、0.05%、0.1%、0.15%(v/v)甲酸的5 mmol/L甲酸铵溶液-乙腈体系,发现加入甲酸后,虽然3种目标分析物的响应强度呈小幅上升,但坎地沙坦酯和拉西地平的峰又出现了重叠。故选择5 mmol/L甲酸铵溶液-乙腈作为最佳流动相。

**图1:**
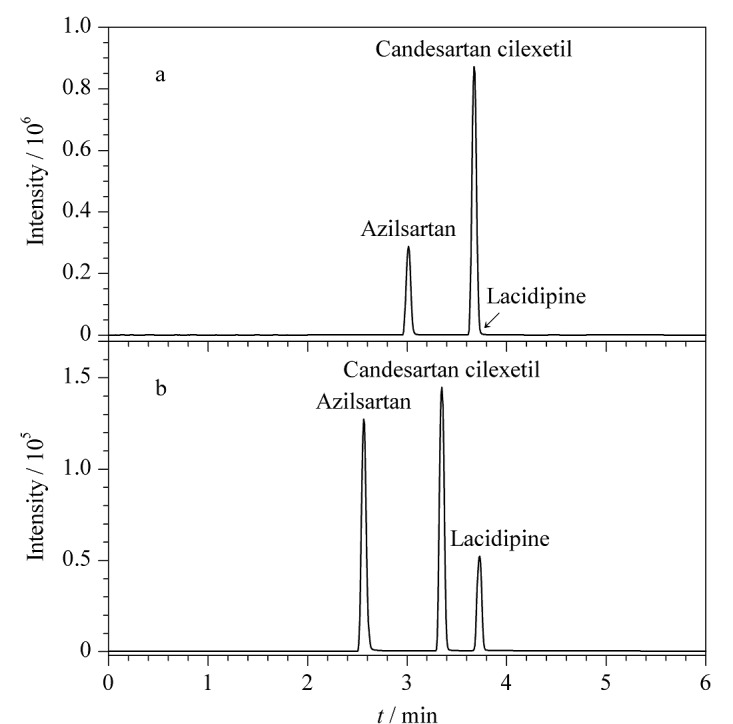
不同流动相对3种降压药分离效果的影响

### 2.2 质谱参数的优化

3种降压药含有氨基、含氮杂环结构、羧基等官能团,在电喷雾离子源下,均可形成分子离子峰[M+H]^+^或[M-H]^-^,实验比较了3种降压药在正、负两种电离模式下的药物前体及产物离子的灵敏度,发现阿齐沙坦和坎地沙坦酯在正离子模式下的响应强度远远高于负离子模式,拉西地平在两种电离模式下的响应强度相当。选择ESI^+^下的分子离子峰作为母离子,进行二级质谱扫描(图2),优先选择*m/z*大、响应强度高且干扰小的碎片离子作为子离子,在MRM模式下检测(见表1)。

**图2:**
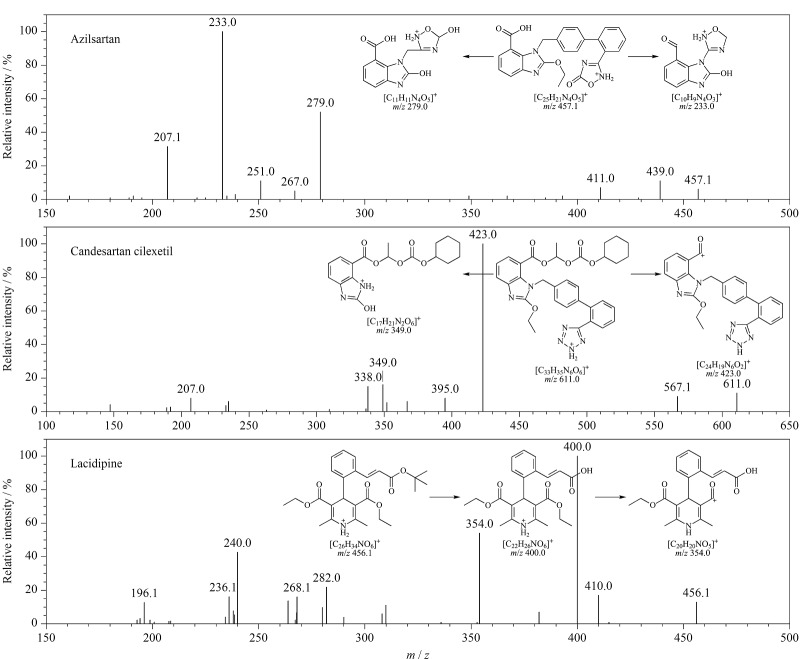
3种降压药的二级质谱图

### 2.3 前处理条件的优化

#### 2.3.1 提取溶剂的选择

基于12种样品基质的形态和3种降压药的极性,文献多以甲醇、乙腈提取^[13,14,23]^。本文选取压片糖果、代用茶、茶饮料3种代表性基质,分别考察了甲醇、乙腈、一定体积比(5∶95、10∶90、20∶80、30∶70、40∶60)的水-甲醇和一定体积比(5∶95、10∶90、20∶80、30∶70、40∶60)的水-乙腈的提取效果。结果显示,甲醇和乙腈的提取效果均较好,水-甲醇和水-乙腈均较差。于是选择甲醇和乙腈依次试验其余基质,结果表明,乙腈对硬胶囊和软胶囊的提取回收率较低,甲醇对配制酒、茶剂、硬胶囊、软胶囊的提取回收率更低;乙腈比甲醇适用的基质多,但仍然不能满足全部基质,因此实验进一步比较了含不同体积分数(0、0.1%、0.2%、0.5%、1%、2%)甲酸的乙腈的提取效果。结果如图3所示,当采用0.2% FA-乙腈提取时,阿齐沙坦在硬胶囊和软胶囊中提取回收率由小于40%提升至大于80%,而在压片糖果、代用茶和饼干中部分目标分析物的回收率有些许降低。为兼顾硬胶囊与软胶囊2种基质,最终选择0.2%甲酸-乙腈作为提取溶剂。

**图3:**
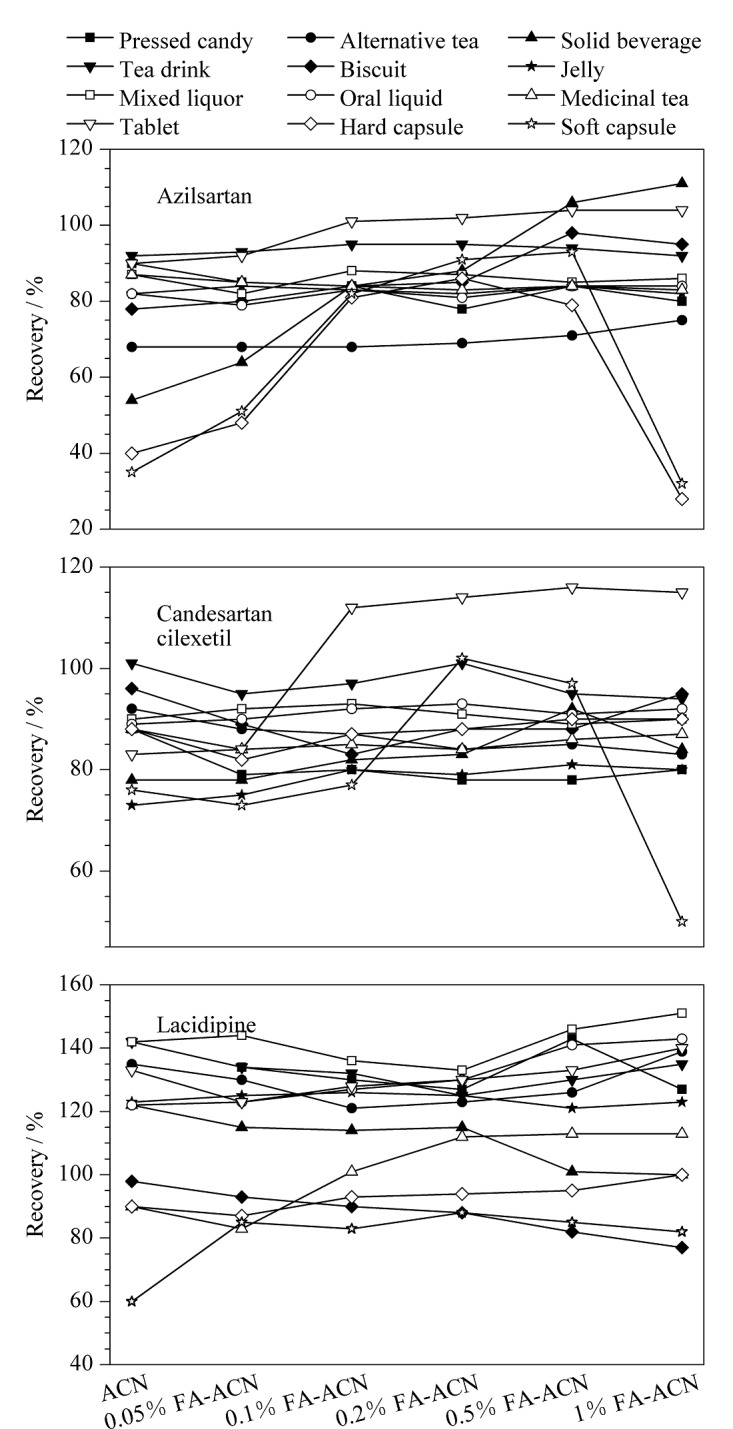
不同提取溶剂对12种基质中3种降压药回收率的影响(*n*=3)

#### 2.3.2 净化条件的选择

本文研究的12种基质中,代用茶基质相对较复杂,主要含有色素、脂肪、糖类等,不同配料的代用茶基质对目标分析物的基质效应(ME)差别较大,如含有决明子的代用茶基质抑制显著。基于3种降压药的p*K*_a_(2~7)和极性,实验考察了Waters Oasis MCX和Waters Oasis HLB小柱的净化效果。结果显示,采用MCX小柱时,拉西地平的净化效果较差,回收率小于60%;而在采用的众多规格的HLB小柱中,200 mg/6 mL小柱的回收率相对较高(80%~85%),但仍未取得较理想的净化回收率。

实验进一步采用QuEChERS方式净化样品,考察了不同配比的吸附剂填料组合(PSA、C_18_和GCB)及少量无水硫酸镁对样品基质的净化效果(图4)。结果显示,随着PSA添加量的增加,阿齐沙坦净化回收率呈快速下降趋势,可能因为阿齐沙坦含有羧基,易被PSA吸附;GCB可去除较多色素^[28]^,净化后的溶液接近无色,但同时3种目标分析物回收率也明显降低;C_18_对目标分析物的回收率均较好。因此选择30 mg、50 mg C_18_分别试验其余基质。结果表明,阿齐沙坦和拉西地平的回收率均较好;坎地沙坦酯在不同基质中的回收率波动较明显,30 mg比50 mg C_18_适用的基质更多。由于含有决明子的代用茶或茶剂经C_18_净化后基质效应明显改善,其余基质在净化前后的基质效应差别并不明显,故当样品为代用茶和茶剂时,进一步净化,净化材料为30 mg C_18_+100 mg无水硫酸镁。

**图4:**
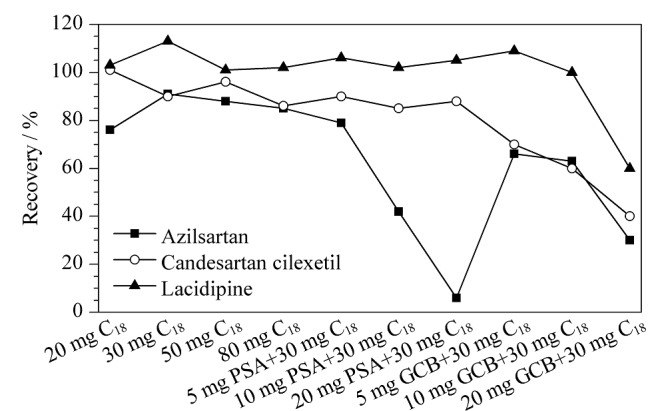
不同配比的吸附剂对3种降压药回收率的影响(*n*=3)

### 2.4 基质效应

基质效应是指在测定过程中,由于目标分析物的离子化效应被样品基质改变,从而使响应信号受到增强或抑制的现象。本文采用标准曲线法评估目标分析物在12种基质中的基质效应,ME=(基质匹配标准曲线斜率/溶剂标准曲线斜率-1)×100%。ME=0表示没有基质效应,ME>0表示基质呈增强作用,ME<0表示基质呈抑制作用。当|ME|≤20%时,则基质效应较弱,在实际检测中可以忽略;当20%<|ME|≤50%或|ME|>50%时,则基质效应中等或较强^[29,30]^。实验结果(图5)显示,阿齐沙坦在代用茶、片剂、硬胶囊和软胶囊等4种基质中基质抑制效应中等或较强,含决明子的代用茶在净化前后基质效应改善明显;坎地沙坦酯受基质影响较弱,基质效应可忽略;拉西地平除了在固体饮料、饼干、硬胶囊和软胶囊4种基质中的基质效应可忽略外,在其余8种基质中基质增强效应中等或较强。为提高定量结果的准确性,本文使用空白基质匹配标准曲线定量以消除基质效应。

**图5:**
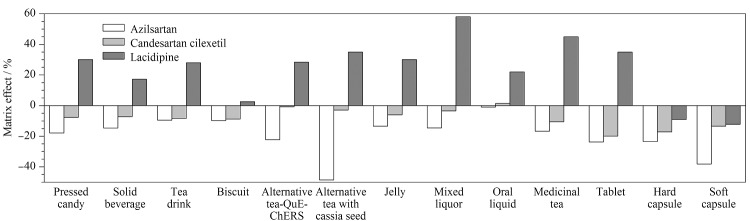
不同样品基质中3种降压药的基质效应(*n*=3)

### 2.5 方法学考察

#### 2.5.1 检出限、定量限和线性范围

采用本方法测定系列基质匹配标准溶液,以各目标分析物的峰面积(*y*)为纵坐标,质量浓度(*x*)为横坐标,绘制标准曲线(见附表1),阿齐沙坦和坎地沙坦酯在0.25~20 μg/L、拉西地平在0.5~40 μg/L范围内线性关系良好,线性相关系数(*r*^2^)均大于0.996。依据GB 5009.295-2023,采用空白基质加标的方法进行检出限和定量限的测定。选取空白样品基质20个平行样,分别添加估算检出限浓度的目标分析物,测定信噪比(*S/N*)和检出概率。以*S/N*≥3、检出概率不低于95%时的浓度确定检出限。选取空白样品基质6个平行样,分别添加定量限浓度的目标分析物,以*S/N*≥10且正确度和精密度均满足要求时确定定量限。结果表明,阿齐沙坦和坎地沙坦酯的检出限为0.01 mg/kg,定量限为0.02 mg/kg,拉西地平的检出限为0.02 mg/kg,定量限为0.04 mg/kg。

#### 2.5.2 加标回收率和精密度

选择压片糖果、代用茶等12种空白样品进行加标回收试验,分别添加低(定量限)、中、高3个加标水平,每个加标水平平行测定6次(*n*=6),计算加标回收率和相对标准偏差(见附表2)。3种目标分析物在12种基质中的平均回收率为86.6%~107.5%,相对标准偏差为1.1%~10.9%,表明方法的正确度和精密度良好。

#### 2.5.3 试样溶液的稳定性

取12种空白基质样品,分别制备定量限时质量浓度的基质样品加标试液,置于室温下,与配制的标准曲线溶液同时进样测定,每个时间点(0、4、8、16、24、48、72 h)平行测定3次。结果显示(图6),试样基质溶液中3种目标分析物在7个时间点测定浓度的RSD(*n*=7)均小于15%,符合GB 5009.295-2023的要求,表明试样溶液在72 h内稳定性良好。但不同样品基质对目标分析物的影响不一致,且拉西地平遇光不稳定^[8]^,建议在48 h内完成分析。

**图6:**
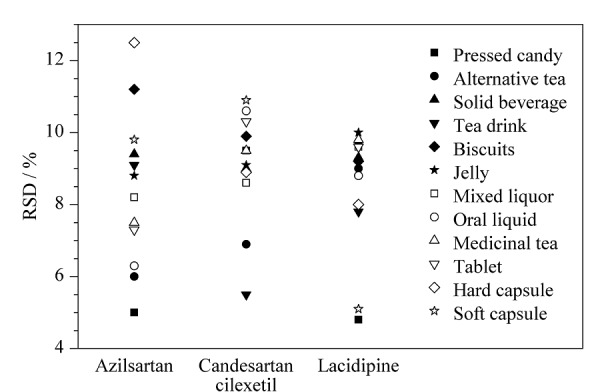
试样溶液在72 h内测定值的RSD(*n*=7)

### 2.6 实际样品检测

为评价该方法的有效性,利用所建立的方法对208个样品进行了检测,阳性样品的测定结果见表2。从目标分析物来看,3种降压药均有检出,检出率最高的药物是阿齐沙坦,含量参差不齐。从样品类型来看,压片糖果、固体饮料和代用茶均检出目标分析物,尤其是宣称具有降压功效的压片糖果,检出率高达20%,应引起重点关注并加强监管力度。

**表2:** 阳性样品的测定结果(*n*=2)

Sample type	Azilsartan	Candesartan cilexetil	Lacidipine
Pressed candy 1	14.8	163	388
Pressed candy 2	23.6	3880	3500
Pressed candy 3	126	-	-
Pressed candy 4	-	-	265
Pressed candy 5	335	-	-
Alternative tea	-	97.5	-
Solid beverage	69.8	-	-

-: not detected.

## 3 结论

本研究基于酸化乙腈提取、QuEChERS净化及超高效液相色谱-串联质谱法建立了一种同时测定压片糖果、固体饮料、代用茶、茶饮料、饼干、果冻、配制酒等普通食品及保健食品(口服液、茶剂、片剂、硬胶囊、软胶囊)中3种降压药的分析方法。方法详细比较了目标分析物在不同流动相中的分离度及响应强度,同时研究了不同样品基质中提取溶剂、净化方式、基质效应及样品试液的稳定性等对结果准确度的影响,覆盖基质类型广泛,并针对不同样品基质类型采用QuEChERS方法净化,提升分析的准确性。该方法用于实际样品检测,结果较为可靠,且操作简单快速,灵敏度高,可为功能宣称食品的非法添加检测和研究提供新思路和新方法,并为市场监管部门的风险监测和日常防控提供了技术依据。
